# Towards a Healthy Diet in an Adolescent Population: The Mediating Role of Motivation and Perceived Barriers between Self-Efficacy and Weight Regulation

**DOI:** 10.3390/healthcare12141454

**Published:** 2024-07-22

**Authors:** María Marentes-Castillo, Isabel Castillo, Inés Tomás, Octavio Álvarez

**Affiliations:** 1 Department of Social Psychology, University of Valencia, 46010 Valencia, Spain; maria.marentes@ext.uv.es (M.M.-C.); octavio.alvarez@uv.es (O.Á.); 2 National Council for Humanities, Science and Technology, Mexico City 03940, Mexico; 3 Department of Methodology of the Behavioral Sciences, University of Valencia, 46010 Valencia, Spain; ines.tomas@uv.es

**Keywords:** self-efficacy, motivation, barriers, healthy eating, healthy weight, adolescents

## Abstract

(1) Background: Adolescence is a critical period when dietary choices are a major concern. It is therefore important to understand the psychological factors that explain these choices. The objective of this study was to assess the predictive role of perceived self-efficacy for healthy eating and weight regulation on healthy and unhealthy eating behavior through the mediation of autonomous motivation, controlled motivation, amotivation, and perceived barriers to daily mechanisms affecting healthy consumption. (2) Methods: A total of 994 adolescents between 15 and 19 years old from Mexico and Spain participated in the study. The Spanish versions of the healthy eating and weight self-efficacy questionnaire, the behavioral regulation in exercise questionnaire, the barriers to healthy eating scale, and the weight-related behaviors scale questionnaire were used to measure the variables of interest. (3) Results: Mediated serial regression analysis showed that barriers to healthy eating (the daily mechanism of food consumption) reduced healthy eating choices. Healthy eating and weight self-efficacy also showed a positive significant relationship with autonomous motivation and a negative relationship with controlled motivation and amotivation. All the types of motivation showed a positive relationship with barriers to healthy eating. (4) Conclusions: The results point out the need to enhance self-efficacy, promote autonomous motivation, and reduce perceived barriers in young people with respect to healthy eating.

## 1. Introduction

Unhealthy eating habits are a significant contributor to global morbidity rates [1]. Malnutrition, including obesity, increases the risk of non-communicable diseases (NCDs) such as heart disease, stroke, diabetes, and some cancers [2]. Adolescence is marked by the prevalence of eating junk food, sugar-sweetened beverages, and foods high in energy [3]. Unhealthy lifestyles caused approximately 11 million deaths worldwide in 2017 [4]. Furthermore, in Mexico, 24.7% of adolescents are overweight and 18% are obese, according to the Government of Mexico [5]. In Spain, the OECD [6] reports that 34.1% of the population aged 5–19 years is overweight or obese. Research on the co-occurrence of these eating behaviors among adolescents is crucial, as eating behaviors throughout adolescence are predictive of behaviors during adulthood and the risk of chronic diseases [7,8].

Adolescence is a critical period of development during which there are significant changes in body shape and weight, as well as notable physical and biological transformations. As adolescents gain greater autonomy and freedom, they become more prepared to assume greater responsibility and participate in decisions about their eating habits [9,10]. These changes can impact other psychological processes, such as body dissatisfaction, concern about their weight, low self-esteem, and the risk of depression and anxiety [11,12]. During the transition from adolescence to early adulthood, weight control behaviors and unhealthy diets are frequently adopted by adolescents, particularly girls, instead of making healthy lifestyle changes. This leads to a decrease in diet quality [11,13,14]. Several studies by Neumark-Sztainer et al. [15,16,17] have shown that adolescents who engage in unhealthy weight control behaviors, such as fasting, skipping meals, or eating too little food, often perceive themselves as overweight. This perception may predict weight status, binge eating episodes, and extreme weight control behaviors. Furthermore, the consumption of unhealthy food among adolescents has been linked to the potential development of depression, anxiety, eating disorders, and mental health issues [16,18]. This highlights the importance of identifying potential factors influencing the adoption of healthy and unhealthy weight control behaviors among this population.

Research on the relationship between psychological factors and healthy eating in adolescence has been limited, with studies primarily focusing on variables associated with behavior such as self-esteem, depression, and body dissatisfaction. However, these studies have produced contradictory results and have not identified which psychological factors can explain the adoption of healthy behavior [12,19,20]. On the other hand, studies that focus on healthy eating and weight regulation in adults aim to understand the factors that lead to the adoption of healthy behaviors and the abandonment of unhealthy ones. Teixeira et al. [21,22], Silva et al. [23], and Ntoumanis et al. [24] investigated motivation as a variable regulating eating and weight regulation, highlighting autonomous motivation as a strong predictor of healthy behavior adoption.

Marentes-Castillo et al. [25,26] tested a predictive model to determine whether grit personality can explain the stage of change towards weight control and the quality of weight-related behavior through the mediation of motivational types. Exploring psychosocial variables that may be associated with a decrease in diet quality and weight regulation in adolescents is an important step towards understanding eating behavior during adolescence and proposing possible avenues for intervention as promotion and prevention measures. However, to our knowledge, little research has been conducted on the psychosocial variables associated with this healthy behavior in adolescents.

A variable related to healthy behavior is perceived self-efficacy, which refers to a person’s beliefs about their ability to organize and execute the necessary actions to achieve a goal. Self-efficacy can be influenced by other variables such as motivational regulation, thought processes, affective states, or external conditions [27]. Self-efficacy is an important variable associated with various health behaviors [28] and an important behavior change indicator due to its influence on initiating specific behaviors [29]. In the field of nutrition, self-efficacy has been associated with improved diet quality, abstinence from unhealthy foods [30,31,32], and enhanced diabetes self-care [33]. Self-efficacy is an important process related to the person’s beliefs about their own capacities. Consequently, if an individual believes they can successfully manage their behavior through their capabilities, they are more likely to perform it [34]. Berman [35] explains that perceived efficacy in weight control can be exerted through resisting unhealthy foods and regulating emotions and physical dissatisfaction, as well as food availability and social pressure from others.

Bandura [36] stated that self-efficacy influences human health at two levels. The first level relates to the ability to cope with biological stressors that mediate health and illness. The second level is associated with direct control over health habits throughout an individual’s life. Therefore, a high level of self-efficacy may strongly predict health-related behavior in adult populations, including weight loss and adherence to a healthy diet [37,38]. However, there has been little research on these associations in adolescents. Studies indicate that self-efficacy is crucial in increasing motivation to adopt healthy eating habits [39] and commitment to physical activity [40]. Self-efficacy for eating more fruits and vegetables is positively correlated with fruit and vegetable intake in teenagers [41,42]. Conversely, self-efficacy for consuming fewer energy-dense snacks is negatively correlated with snack intake [41].

Self-efficacy and motivation have been found to be associated with health behaviors, but they are independent factors [22,23,38]. Bandura [27] suggests that efficacy beliefs are one of many determinants that regulate motivation, affect, and behavior. Einserberg et al. [28] suggest that an increase in controlled motivation may lead to unhealthy eating behaviors due to the perceived inability to conform to social standards of beauty, eating, and thinness [43,44], which may be associated with a low sense of self-efficacy. In this line, Einserberg et al. [28] found that controlled motivation was positively associated with the presence of eating disorder symptoms, and this association was stronger among adolescents with a low sense of self-efficacy, while autonomous motivation did not show a significant association.

This research focuses on external barriers in the domain of eating that may hinder healthy consumption in the adolescent population [45] as opposed to individual and social barriers [46]. Environmental constraints can hinder engagement in adolescent health behavior by acting as perceived barriers to change, such as the cost of food, lack of time, and availability.

**Figure 1:**
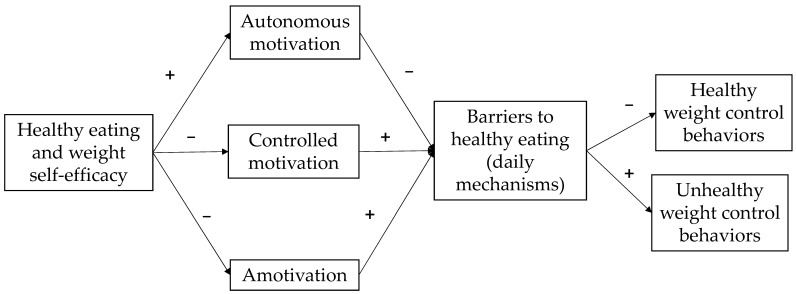
Serial mediation model for healthy eating and weight self-efficacy on healthy and unhealthy weight control behaviors through types of motivation and perceived barriers to healthy eating (daily mechanisms).

Based on the previous literature, and to expand our knowledge in the promotion of healthy and sustainable diets, the purpose of this study was to evaluate the predictive role of perceived self-efficacy for healthy eating and weight regulation on healthy and unhealthy eating behavior for weight control in an adolescent population (aged 15 to 19 years) from Mexico and Spain. This study used a serial mediation of autonomous motivation, controlled motivation, and amotivation, as well as perceived barriers to daily mechanisms of healthy eating (see Figure 1).

## 2. Materials and Methods

### 2.1. Participants

A total of 994 adolescents from Mexico (n = 668) and Spain (n = 326) participated in the study, of whom 597 were females, 390 were males, and 7 were of an unspecified gender. Participants were selected by non-probability cluster sampling from different schools in Mexico and Spain and ranged in age from 15 to 19 years (M = 16.53; SD = 1.18). The participants included in the study were required to be enrolled in the academic year with regular attendance and aged between 15 and 19 years.

### 2.2. Instruments

The healthy eating and weight (HEW) self-efficacy [47] scale, adapted to the Spanish version [48], measures the belief about the ability to engage in healthy eating. It consists of 11 items on a Likert-type scale from 1 to 5 (strongly disagree to strongly agree). It contains 2 factors: self-efficacy towards healthy consumption with 7 items (e.g., “I have confidence that I can attain and maintain my ideal weight”) and healthy weight maintenance with 4 items (e.g., “I am usually confident that I can reach and maintain my ideal weight”). For this study, a composite score reflecting global self-efficacy was used. The fit for a second-order factor CFA model showed acceptable values (CFI = 0.94; TLI = 0.93; RMSEA = 0.06).

The behavioral regulation in exercise questionnaire (BREQ-3) adapted to weight control [49] in the Spanish version [25] was used. This questionnaire measures the different types of motivation regulations towards weight control behavior. It consists of 23 items on a Likert-type scale ranging from 1 to 5 (not at all true to completely true). The questionnaire measures intrinsic, integrated, identified, introjected, and external regulation, and amotivation. Intrinsic, integrated, and identified regulation can be grouped into autonomous motivation, which is composed of 11 items (“because controlling my weight is a fundamental part of who I am”). Introjected and external regulation is grouped into controlled motivation with 8 items (“because I feel guilty when I don’t do it”), and amotivation with 4 items (“I don’t see the point in controlling my weight”) remains the same. The fit for a 3-factor CFA model was acceptable (CFI = 0.95; TLI = 0.94; and RMSEA = 0.07).

The barriers to healthy eating (BHE) [46] adapted to the Spanish version [48] assesses the frequency of perceived barriers to healthy eating and weight control. It comprises 22 items rated on a Likert scale ranging from 1 to 5 (not a problem at all to a very important problem). The items are divided into three factors: self-control and motivation (12 items), daily mechanisms (7 items), and social support (3 items). Out of the 22 items, we have only used 7 that pertain to the daily mechanisms factor (“I find it difficult to select the right foods when shopping”). The fit of the 3-factor CFA model for this scale was acceptable (CFI = 0.91; TLI = 0.90; RMSEA = 0.06).

The weight-related behaviors scale [50] adapted to the Spanish version [25] assesses the weight loss behaviors of individuals, both healthy and unhealthy. It comprises 15 items on a Likert-type scale ranging from 1 to 5 (never to always), divided into healthy weight control behaviors with 6 items (“consume less sugar”) and unhealthy weight control behaviors with 9 items (“consume very little food”). The fit indices for this scale were acceptable (CFI = 0.94; TLI = 0.92; and RMSEA = 0.07).

### 2.3. Procedure

This study was conducted in accordance with international ethical guidelines consistent with the American Psychological Association and in accordance with the guidelines established in the Declaration of Helsinki. All procedures involving participants in the research study were approved by the Experimental Research Ethics Committee of the University of Valencia (Ref: 1707311).

The data collection consisted of four stages. Firstly, we contacted 9 schools in Mexico and 12 schools in Spain, requesting permission to collect data online from adolescents between the ages of 15 and 19 who were active in the school year. All schools agreed to participate in the study. Secondly, after obtaining informed consent from parents of adolescents under 16 years of age, we distributed the online survey to all school participants. During stage 3, the survey was closed and a total of 1097 students from 9 schools in Mexico and 9 schools in Spain participated. In stage 4, we analyzed 994 participants for the present study after applying the inclusion and exclusion criteria. Data collection took place from March to June 2022 using online Google Forms. Before data collection, a real-time connection was established to explain the procedure to the participants and inform them of the presence of the teacher in charge of the school group. All participants consented to be part of the study and were informed of their voluntary and anonymous participation in the study and spent 15–20 min filling out online forms.

### 2.4. Data Analysis

Descriptive and correlational analyses were conducted for the total sample and by country to examine the relationships between the study variables. The percentage of missing data in the present study was less than 5% [51], indicating that it is unlikely to be a significant issue. To compare the sample studied by gender and country, a hypothesis test was carried out to evaluate the differences between correlation coefficients using Fisher’s z-statistic. The association between HEW self-efficacy and the frequency of weight-related behaviors was tested, as well as the mediating role of types of motivation (autonomous, controlled, and amotivation) and barriers to healthy eating in this relationship, using Model 80 in SPSS macro-PROCESS version 3.4.1 [52]. Two separate models, one for each of the outcomes (healthy and unhealthy weight control behaviors), were run. The analysis included the examination of direct and indirect effects between variables and coefficients of determination (R^2^). The statistical significance was set at 0.05. The significance of the indirect effects (IEs) was tested using 95% bootstrap confidence intervals, with 5000 replications [52]. The indirect effects were considered significant when the confidence interval did not include zero, supporting a mediation effect.

## 3. Results

Table 1 shows the means, standard deviations, skewness, kurtosis, and internal reliability of the study variables. The normality of the data shows skewness values between −0.27 and 1.40, and kurtosis values between −0.56 and 2.66. The adolescent sample reported perceiving themselves with self-efficacy to regulate their eating and weight (above the scalar mean), as well as autonomously motivated, but also in a controlled way to regulate weight. On average they can perceive barriers to the daily mechanisms of their consumption and reported performing healthy and unhealthy behaviors to regulate weight below the mean. The internal reliability coefficients for all the study variables were satisfactory.

The correlations among the study variables were statistically significant and aligned with the expected direction. Highlighting those to be tested in the hypothesized model, HEW self-efficacy correlated positively with autonomous motivation and negatively with amotivation. Autonomous motivation was negatively correlated with perceived barriers to daily mechanism eating. Controlled motivation and amotivation were positively correlated with perceived barriers to daily mechanism eating. And finally, perceived barriers were positively related to unhealthy behavior and negatively related to healthy weight control behavior (see Table 2).

The gender-based correlations showed statistically significant differences only for HEW self-efficacy with autonomous motivation, and with perceived barriers to healthy eating in daily mechanisms with stronger correlations in the female group. This suggests that higher levels of HEW self-efficacy are associated with higher levels of autonomous motivation and lower perceptions of barriers to daily mechanism eating. On the other hand, significant differences were found in the correlation between amotivation and barriers to daily healthy eating. The correlation was stronger for men, indicating that the greater the amotivation, the greater the perception of barriers to healthy daily mechanism eating (see Table 3).

There were significant differences between countries in the correlation of HEW self-efficacy with autonomous motivation, autonomous motivation with perceived barriers to healthy eating in daily mechanisms, and perceived barriers with healthy weight control behaviors. The Mexican sample consistently exhibits the highest correlation values, suggesting that greater HEW self-efficacy leads to increased autonomous motivation. Similarly, greater autonomous motivation is associated with fewer perceived barriers to healthy eating, while higher perceived barriers are linked to less frequent engagement in healthy weight control behaviors (see Table 4).

**Figure 2:**
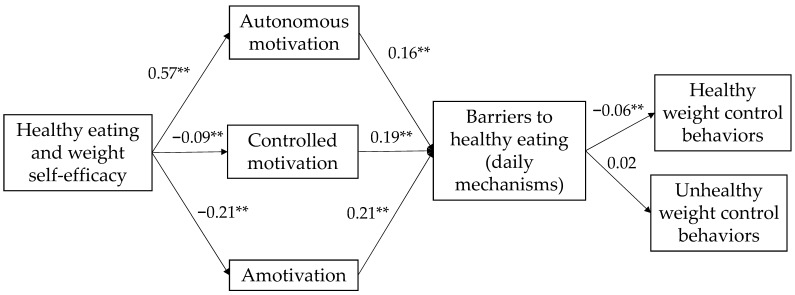
Unstandardized solution of serial mediation model. ** *p* < 0.01.

The proposed model included gender and country variables as control variables to account for significant gender and country differences. To assess the proposed model (see Figure 1), we conducted a serial mediation analysis. We tested whether the types of motivation and perceived barriers to healthy eating mediate the effect of HEW self-efficacy on healthy and unhealthy weight regulation behaviors. Figure 2 shows that types of motivation (autonomous, controlled, and amotivation) and perceived barriers to healthy eating mediated the relationship between HEW self-efficacy and healthy and unhealthy weight regulation behaviors (see Table 5). The results of the model indicated that HEW self-efficacy explained the following percentages of variance: 32% for autonomous motivation, 2% for controlled motivation, and 5% for amotivation. Additionally, HEW self-efficacy explained 32% of the variance of perceived barriers to daily mechanism eating, 24% of healthy weight control behaviors, and 16% of the variance of unhealthy weight control behaviors. All percentages were statistically significant.

Table 6 shows that the relationship between HEW self-efficacy and healthy weight control behaviors is significant only through controlled motivation, amotivation, and perceived barriers to healthy eating as a simple mediation, and as a serial mediation through autonomous motivation and perceived barriers to healthy eating. The relationship between HEW self-efficacy and unhealthy weight control behaviors is significant only through controlled motivation as a simple mediation.

## 4. Discussion

Adolescent dietary choices are a major concern for health professionals, but the reasons for unhealthy adolescent diets are not well understood. The purpose of this study was to explore psychological factors associated with healthy and unhealthy eating behaviors, specifically, whether HEW self-efficacy can predict healthy and unhealthy weight control behaviors through the serial mediation of motivation (autonomous, controlled, and amotivation) and perceived barriers to healthy eating.

The study’s findings confirm that HEW self-efficacy and motivation are factors associated with healthy eating behaviors, as previously suggested by Teixeira et al. [22] and Silva et al. [23]. Motivation is a significant factor in healthy behavior, including eating and weight regulation [22,25,26]. Therefore, we hypothesized that motivation could mediate the relationship between HEW self-efficacy and healthy and unhealthy weight control behaviors. Furthermore, we examined perceived barriers to healthy eating, specifically exploring the daily mechanisms factor, to determine if external factors hinder the frequency of healthy consumption choices. The examined model’s results have shown direct and significant relationships between HEW self-efficacy and autonomous motivation. Additionally, there were direct, negative, and significant relationships between HEW self-efficacy and controlled motivation and amotivation. These findings confirm that self-efficacy plays a key role in enhancing motivation [39].

The study also found direct relationships between types of motivation and perceived barriers to daily mechanisms. Amotivation showed the strongest relationship with perceived barriers, followed by controlled motivation and autonomous motivation. However, perceived barriers to daily mechanisms only weakly negatively correlated with healthy weight control behaviors and showed no significant relationship with unhealthy weight control behaviors. The indirect effects of the model indicate that HEW self-efficacy can explain healthy weight control behaviors, but only through controlled motivation, amotivation, and perceived barriers to daily mechanisms. However, these factors only act as simple mediators of healthy weight control behaviors. As a serial mediation, the relationship only is significant through autonomous motivation and perceived barriers. This relationship indicated that whether HEW self-efficacy impacts autonomous motivation, perceived barriers can hinder and reduce the possibility of choosing a healthy behavior. On the other hand, HEW self-efficacy can only predict unhealthy weight control behaviors through controlled motivation, again as a simple mediator.

Although HEW self-efficacy can predict both healthy and unhealthy weight control behaviors, it is important to note that controlled motivation primarily mediates these relationships, particularly for unhealthy weight control behavior. This finding confirms that self-efficacy can be a function of other variables such as motivation regulation or external conditioning factors that also influence behavior [27].

Autonomous motivation has been shown to mediate healthy weight control behavior in adults [24,25,26], but not in adolescents. Marentes-Castillo et al. [53] showed that controlled motivation also contributes to failure in cognitive self-control when adolescents choose consumer products, such as junk food, sugar-sweetened beverages, and foods high in energy [3]. Thus, controlled motivation appears to play a primary role in the eating behaviors of adolescents. According to Einserberg et al. [28], controlled motivation increases the risk of unhealthy eating behaviors and disorders in young people due to the perceived inability to control their behavior [43,44].

However, while perceived barriers to change can be a barrier to health behavior engagement [45], their role in our proposed model is not entirely clear. On one hand, the strength of controlled motivation for choosing a consumer product may outweigh any barriers, as demonstrated in our model and reported by Marentes-Castillo et al. [53]. On the other hand, it appears that daily consumption mechanisms, such as knowing which foods to eat to lose weight or reduce calories and fat, are not perceived as barriers for consumers. It is important to note that the perceived barriers were measured using an instrument designed for adults. This instrument inquired about the mechanisms for acquiring and preparing food, as well as availability at home, among other factors. As such, these barriers may not be relevant for adolescents, as they do not have direct control over these factors [27]. For this reason, future research should investigate the barriers that only young people perceive, despite being the ones who decide on the choice, purchase, and consumption of goods.

This study demonstrated that HEW self-efficacy plays a crucial role in enhancing motivation and can influence changes in eating habits, similar to previous research about the relationship between self-efficacy and eating habits in adolescents [41,42]. Self-efficacy acts as one of the determinants that regulate motivation [27,39]. However, its impact can be weakened by other factors such as controlled motivation and barriers that hinder the desired action [27]. Therefore, strengthening the belief in the effectiveness of consuming a healthy diet and regulating weight can increase autonomous motivation. This means that young people can choose to consume a healthy diet by their own will and make decisions that lead to more frequent healthy weight control behaviors. Although HEW self-efficacy may reduce controlled motivation and amotivation, perceived barriers will still be present and ultimately impact healthy weight control behaviors. Finally, the relationships found for healthy weight control behaviors do not seem to apply to unhealthy weight control behaviors. It is important to note that the choice to engage in unhealthy behaviors to regulate weight is not associated with or explained by efficacy belief, but rather can occur in the presence of controlled motivation.

The proposed model can be a valuable contribution to our understanding of healthy and unhealthy behavior among young adolescents. It leads us to affirm that self-efficacy and motivation play a relevant role in understanding the eating habits of this population. The proposal to promote healthy diets aims to promote and enhance individual elements such as personal efficacy and autonomous motivation as determining factors in the choice of healthy consumer products. It is important to pay attention to the psychological factors that explain the eating choices of adolescents, and not only the choices themselves.

Future suggestions include introducing additional variables, such as personality and self-control, clarifying the role of perceived barriers in young adolescents, and including families. In addition, more research is needed on the types of barriers to eating that adolescents perceive. Interventions can improve self-efficacy for healthy eating and weight regulation by teaching adolescents to make better food choices, leading to greater autonomy in decision-making and potentially reducing perceived barriers to healthy eating.

The study has limitations, including differences in participants between the Mexican and Spanish groups (although this effect was mitigated by introducing country as a control variable), and the cross-sectional nature of the study, which precludes causal conclusions. Additionally, the use of the barriers to healthy eating as the original tool for adults represents a limitation. In future studies, the instrument will be adapted to identify the specific barriers that adolescents perceive regarding their eating. Further exploration of additional variables may provide greater clarity regarding the pathway to healthy and unhealthy eating.

## 5. Conclusions

This study represents a significant advance in the field of adolescent psychology, offering a novel approach to understanding the relationship between eating habits and health behaviors. Our findings confirm that integrating a psychological perspective, emphasizing the efficacy of eating healthy and regulating weight, is an effective starting point for promoting health behaviors in adolescents. To achieve optimal outcomes, this type of intervention must involve the expertise of both a psychologist specializing in health behavior and a nutritionist who can provide a comprehensive, psychological-focused approach to dietary guidance.

## Figures and Tables

**Table 1:** Descriptive statistics and reliability of the study variables (n = 994).

Variables	Mean	SD	Skewness	Kurtosis	Alpha
HEW self-efficacy	3.44	0.85	−0.26	−0.44	0.89
Autonomous motivation	3.27	0.94	−0.27	−0.52	0.91
Controlled motivation	2.35	0.96	0.43	−0.56	0.86
Amotivation	1.83	0.89	1.07	0.56	0.78
Barriers to healthy eating	2.60	0.90	0.18	−0.49	0.83
Healthy WC behaviors	2.48	0.58	0.28	0.20	0.72
Unhealthy WC behaviors	1.53	0.52	1.40	2.66	0.61

Note: All range variables = 1–5, HEW = healthy eating and weight; WC = weight control.

**Table 2:** Bivariate correlations of the study variables (n = 994).

Variables	1	2	3	4	5	6
1. HEW self-efficacy						
2. Autonomous motivation	0.53 **					
3. Controlled motivation	−0.08	0.22 **				
4. Amotivation	−0.20 **	−0.25 **	0.29 **			
5. Barriers to healthy eating	−0.42 **	−0.06 **	0.35 **	0.31 **		
6. Healthy WC behaviors	0.43 **	0.32 **	0.07 *	−0.21 **	−0.23 **	
7. Unhealthy WC behaviors	−0.11 *	−0.03	0.35 **	0.18 **	0.17 **	0.12 **

Note: HEW = healthy eating and weight; WC = weight control. * *p* < 0.05; ** *p* < 0.01.

**Table 3:** Results of values of correlation differences by gender for study variables.

Variables Correlated	Male (n = 390) Correlation	Female (n = 597) Correlation	z
HEW self-efficacy—Autonomous motivation	0.47 **	0.57 **	−2.10 *
HEW self-efficacy—Controlled motivation	−0.04	−0.10 *	0.92
HEW self-efficacy—Amotivation	−0.18 **	−0.21 **	0.48
HEW self-efficacy—Barriers HE	−0.30 **	−0.48 *	3.27 **
HEW self-efficacy—Unhealthy WC behaviors	0.42 **	0.45 **	−0.06
HEW self-efficacy—Healthy WC behaviors	−0.09	−0.12 **	0.46
Autonomous motivation—Barriers HE	0.00	−0.10 *	1.53
Controlled motivation—Barriers HE	0.40 **	0.33 **	1.23
Amotivation—Barriers HE	0.39 **	0.25 **	2.39 *
Barriers HE—Healthy WC behaviors	−0.18 **	−0.26 **	1.28
Barriers HE—Unhealthy WC behaviors	0.23 **	0.15 **	1.27
Healthy WC behaviors—Unhealthy WC behaviors	0.13 *	0.10 *	0.47

Note: HEW = healthy eating and weight, HE = healthy eating, and WC = weight control. ** *p* < 0.01, * *p* < 0.05.

**Table 4:** Results of values of correlation differences by country for study variables.

Variables Correlated	Mexico (n = 668) Correlation	Spain (n = 326) Correlation	z
HEW self-efficacy—Autonomous motivation	0.56 **	0.46 **	−1.97 *
HEW self-efficacy—Controlled motivation	−0.09 *	−0.07 *	−0.24
HEW self-efficacy—Amotivation	−0.20 **	−0.18 **	−0.38
HEW self-efficacy—Barriers HE	−0.45 **	−0.35 *	1.78
HEW self-efficacy—Unhealthy WC behaviors	0.45 **	0.37 **	1.36
HEW self-efficacy—Healthy WC behaviors	−0.11 **	−0.12 **	0.12
Autonomous motivation—Barriers HE	−0.13 **	0.02	−2.22 *
Controlled motivation—Barriers HE	0.32 **	0.41 **	−1.53
Amotivation—Barriers HE	0.29 **	0.38 **	−1.49
Barriers HE—Healthy WC behaviors	−0.28 **	−0.14 *	−2.16 *
Barriers HE—Unhealthy WC behaviors	0.17 **	0.19 **	−0.30
Healthy WC behaviors—Unhealthy WC behaviors	0.10 *	0.16 **	−0.90

Note: HEW = healthy eating and weight, HE = healthy eating, and WC = weight control. ** *p* < 0.01, * *p* < 0.05.

**Table 5:** Types of motivation and perceived barriers to healthy eating (daily mechanisms) as mediators between HEW self-efficacy and healthy and unhealthy weight control behaviors.

Dependent Variables Predictors	B	95% LL CI	95% UL CI	R^2^
Autonomous motivation				0.32 ***
Barriers HE	0.57	0.51	0.63	
Controlled motivation				0.02 ***
Barriers HE	−0.09	−0.16	−0.02	
Amotivation				0.05 ***
Barriers HE	−0.21	−0.27	−0.14	
Barriers HE				0.32 ***
Autonomous motivation	0.16	0.10	0.23	
Controlled motivation	0.19	0.14	0.25	
Amotivation	0.21	0.15	0.27	
Healthy WC behaviors				0.24 ***
HEW self-efficacy	0.23	0.18	0.28	
Autonomous motivation	0.04	−0.00	0.09	
Controlled motivation	0.08	0.04	0.12	
Amotivation	−0.08	−0.12	−0.04	
Barriers HE	−0.06	−0.10	−0.01	
Unhealthy WC behaviors				0.16 ***
HEW self-efficacy	−0.02	−0.06	0.02	
Autonomous motivation	−0.03	−0.05	0.01	
Controlled motivation	0.18	0.11	0.16	
Amotivation	0.03	0.01	0.07	
Barriers HE	0.02	−0.02	0.04	

Note: HE = healthy eating, HEW = healthy eating and weight, WC = weight control, B = unstandardized regression coefficient, LL = lower limit, UL = upper limit, CI = confidence interval, and R^2^ = coefficient of determination. *** *p* < 0.001.

**Table 6:** Indirect effects of HEW self-efficacy on healthy and unhealthy weight control behaviors.

Indirect Effect [Mediator]	Indirect Effect	Bootstrap LL 95% CI	Bootstrap UL 95% CI
Healthy WC behaviors			
[Autonomous motivation]	0.025	−0.002	0.052
[Controlled motivation]	−0.007	−0.014	−0.001
[Amotivation]	0.016	0.007	0.026
[Barriers HE]	0.028	0.006	0.051
[Autonomous motivation—Barriers HE]	−0.009	−0.019	−0.001
[Controlled motivation—Barriers HE]	0.001	0.000	0.004
[Amotivation—Barriers HE]	0.004	0.000	0.008
Unhealthy WC behaviors			
[Autonomous motivation]	−0.016	−0.043	0.008
[Controlled motivation]	−0.016	−0.030	−0.003
[Amotivation]	−0.006	−0.016	0.003
[Barriers HE]	−0.010	−0.030	0.009
[Autonomous motivation—Barriers HE]	0.002	−0.001	0.006
[Controlled motivation—Barriers HE]	−0.000	−0.001	0.000
[Amotivation—Barriers HE]	−0.000	−0.002	0.001

Note: HEW = healthy eating and weight, WC = weight control, HE = healthy eating, LL = lower limit, UL = upper limit, and CI = confidence interval.

## Data Availability

All data used in this study are presented in the manuscript.
